# Effective methods for reactivating inactive blood donors: a stratified randomised controlled study

**DOI:** 10.1186/s12889-020-08594-9

**Published:** 2020-04-10

**Authors:** Jian Ou-Yang, Chun-Hua Bei, Hua-Qin Liang, Bo He, Jin-Yan Chen, Yong-Shui Fu

**Affiliations:** 1 Guangzhou Blood Center, 7th F., 31st Luyuan Rd. Yuexiu Dist, Guangzhou, Guangdong China; 2 The Key Medical Disciplines and Specialties Program of Guangzhou, Guangzhou, Guangdong China

**Keywords:** Blood donation, Altruism, Inactive donors, Recruitment, Self-reported deterrents

## Abstract

**Background:**

Recruiting of sufficient numbers of donors of blood products is vital worldwide. In this study we assessed the efficacy and cost-effectiveness of telephone calls and SMS reminders for re-recruitment of inactive blood donors.

**Methods:**

This single-centre, non-blinded, parallel randomised controlled trial in Guangzhou, China included 11,880 inactive blood donors whose last donation was between January 1 and June 30, 2014. The donors were randomly assigned to one of two intervention groups (telephone call or short message service [SMS] communications) or to a control group without intervention. SMS messages with altruistic appeal were adopted in the SMS group; in addition to altruistic appeal, reasons for deferral of blood donation were also asked in the telephone group. All participants were followed up for 1 year. The primary outcome was re-donation rate, and rates in different groups were compared by intention-to-treat (ITT) analysis and estimation of the average treatment effect on the treated (ATT). Secondary outcomes were the self-reported deterrents. Other outcomes included the re-donation interval, and the incremental cost-effectiveness ratio (ICER) of telephone calls and SMS reminders on re-recruitment.

**Results:**

ITT analysis revealed no significant differences in the re-donation rate among the three groups. ATT estimations indicated that among compliers, telephone calls significantly increased re-donation compared to both SMS reminders and no intervention. Donor return behaviour was positively associated with receiving reminders successfully, being male, older age, and previous donation history. The SMS reminder prompted donors to return sooner than no reminder within 6 months, and according to ICER calculations, SMS reminders were more cost-effective than telephone calls. Donors reported time constraints as the most main causes of self-deferral in the telephone group, and altruistic appeal had a positive effect on these donors.

**Conclusions:**

Interventions to reactivate inactive blood donors can be effective, with telephone calls prompting more donors to return but at a greater cost than SMS messages. SMS reminder with altruistic appeal can urge donors to re-donate sooner within 6 months than no reminder.

**Trial registration:**

NCT03366441 (Reactivation of Inactive Blood Donors). Retrospectively registered 4 December 2017.

## Background

Blood products play a vital role in saving lives in a wide variety of medical conditions. Along with the rapid development of the economy and improvement of modern medicine in China, the demand for blood products has continued to grow. Many cities in China have faced a “blood shortage” dilemma, in which the blood supply cannot meet the clinical demand [1]. Therefore, effective strategies for recruiting sufficient numbers of blood donors are critically needed. It has been widely established that repeat donors have a lower transfusion-transmissible infection risk [2], and this reduced risk is maintained in donors who have not donated blood for 5 years [3]. In addition, individuals with a previous donation experience are more likely to restart donations in the future than are first-time donors [2]. The approach of reactivating inactive donors is nonetheless challenging. The percentage of donations from repeat donors in China was reported to be 34–40% [4–6], which is lower than that in the United States (68%) [7] and that in England (55%) [8]. It is essential to determine effective methods for reactivating lapsed donors (defined as those who have made at least one donation within the last 24 months, but not within the previous 12 months) and inactive donors (those who have made at least one donation but have not donated within the previous 24 months) [9] in order to maintain an adequate blood supply.

The most common and accessible reminders for promoting the return of blood donors include telephone calls, cell phone short message service (SMS) messages, mailings, and e-mails, which may help to support the intrinsic motivations of donors, thereby increasing their commitment to donation [10]. Aside from its wide availability, low cost, and convenience, SMS messaging has been proven to be an effective intervention for a variety of health behaviours [11, 12], including blood donation [13, 14]. Upon receiving SMS message, donors may recall positive feelings from previous blood donations, thus increasing their desire to repeat the experience [11]. However, instead of sending only generic information via SMS messages, telephone calls have the advantage of personalizing communication with donors. Godin et al. found that a first phone call reminder could encourage first-time donors to return [15]. Sinclair et al. reported that the use of an adapted motivational interview via telephone calling could increase the chance of future donation [10]. Donors also reviewed their donation experience in consideration of their wider motivations for giving and extended this line of thinking to problem-solving solutions to perceived barriers [10].

Eliminating the deterrents for inactive donors is a critical retention strategy. Research has shown that participants are more likely to donate again after they have been invited to report their reasons for not donating [16]. Reports from different countries have indicated that medical reasons, time constraints, fear (of needles/bleeding), and negative physical reactions are the most frequently self-reported deterrents among lapsed and inactive donors [17–21].

Blood donors report multiple motivations for blood donation [22–26], and campaigns to encourage them to donate should focus on multiple perspectives for different groups. However, in the era of information overload, SMS messaging has the disadvantage of being easily ignored by recipients; hence, the recruitment message needs to be short and simple to understand. Altruistic appeal is a common and acceptable way of recruiting blood donors, which has been widely adopted in various campaigns. Therefore, in the present study, SMS messages with an altruistic appeal that emphasized “saving a life” were sent in an attempt to re-recruit inactive blood donors. Meanwhile, telephone calls were made to re-recruit inactive donors by calling them with not only an altruistic appeal but also questions regarding the reasons why they stopped and providing corresponding solutions.

The objective of this stratified, randomised controlled trials was to assess the efficacy and cost-effectiveness of telephone calls and SMS messages for blood donor re-recruitment. A secondary objective was to explore the self-reported reasons for deferral among donors who received the telephone call. Other aims included evaluating donor return according to demographic characteristics, the time to return among the different interventions, and the cost of telephone calls versus SMS messages to former blood donors.

## Methods

### Study design, setting and participants

The Guangzhou Blood Center is one of the largest blood centres in China along with the Beijing and Shanghai Centers. A total of 263,681 donors donated blood during 2014, of which 179,964 (68.3%) then became inactive and 83,717 (31.7%) donated again before 2016 [27].

**Fig. 1:**
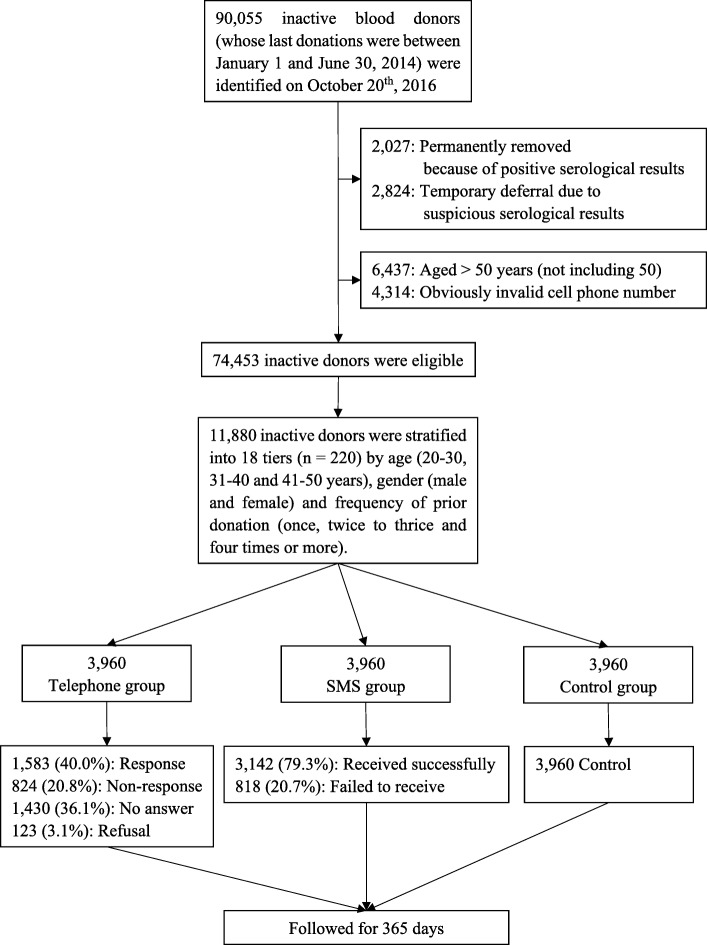
Flow chart of the study design

This single-centre, non-blinded parallel randomised controlled trial involved two intervention groups (telephone or SMS reminders) and a no-intervention control group. Figure 1 shows a flow chart of the study design. During the experiment period, donors who had donated blood after July 1, 2014 were continuing to receive phone call and/or SMS reminders occasionally from Guangzhou Blood Center. Therefore, in order to avoid contaminations, both whole blood and apheresis platelet donors whose last donations were between January 1 and June 30, 2014 were eligible for the screening. The age range for blood donors in mainland China is 18–55 years. Those aged above 50 years old were excluded from the screening based on the previous finding that most older individuals are unlikely to donate again due to physical reasons [28]. All data were provided by the Guangzhou Blood Center through the Blood Donation and Supply System [27].

The calculated sample size needed for each group was 2252 [29], and in order to better detect significant differences and balance the sample size in each tier, the actual sample size was 3960 in each group (11,880 total). All participants were stratified into 18 tiers by age (20–30, 31–40, and 41–50 years), gender (male and female), and frequency of prior donation, which refers to number of times a donor donated before becoming inactive (one time, two or three times, and four times or more). Based on a computer-generated list of random numbers, the first 220 eligible participants in each tier were assigned to the telephone group, the 221st to 440th were assigned to the SMS group, and the 441st to 660th were assigned to the control group.

### Interventions and endpoint

The experimental period lasted from October 20 to November 10, 2016. The details of the recruitment method were described in the pilot study [29]. The start time was set at the day that an intervention was made.

#### Telephone call reminder

Telephone interviews were conducted over a 1-month period by two interviewers (O-Y and BEI, staff members at the Guangzhou Blood Center with the responsibility of blood donor recruitment). The interviews were conducted using pre-designed questionnaires and lasted 2.4–21.3 min (mean ± SD, 4.8 ± 1.2 min). Prior to this study, the interviewers summarized the barriers to donation frequently mentioned by participants in the pilot study [29], discussed the challenges that arose during the interviews, reviewed the optimal response techniques, and practiced via role playing to ensure adherence to the script.

Donors who could not be reached by telephone because the phone number was wrong were marked as non-responders. Donors with a disconnected phone line or who did not answer were called two more times on subsequent days before being classified as “no answer”. Donors who answered the phone but refused the interview request were marked as “refusal”. All participants, including those marked as non-responders, no answer, and refusal, were further followed up as described below. After contact was successfully made, a brief and scripted interview was delivered with the donors’ permission (Additional file 1).

#### SMS reminder

In the SMS group, participants received a text message making an altruistic appeal. As follow:

*“Dear donors,*

*Thank you for your donation through which your love brought hope to those helpless patients and your donated blood reignited the fire in their lives. If you can, please consider donating blood again to save a life.*

*Thank you again for your support!”*

The message was sent via the SMS platform of the Guangzhou Blood Center. Message receipts, which stated if a message was received successfully or not were retrieved from the SMS platform within 48 h. All participants, whether they received the message or not, remained on the list for further follow-up as described below.

#### Follow-up and outcome measures

The donation activity of each participant was followed for 365 days from the recruitment day. All participants could be followed via the Blood Donation and Supply System in which their blood donation records in Guangzhou could be checked. The primary outcome was to identify the occurrence of the first next blood donation attempt among all participants within the 1-year follow-up and evaluate the return rates according to donor characteristics (gender, age and past donation frequency). A participant was classified as a re-activated donor if he/she made at least one subsequent donation by the end of the 1-year follow-up period; otherwise, the donor was classified as “no return”. The secondary outcomes were the main self-reported reasons for deferral given by donors during the telephone calls. Other outcomes included the re-donation interval for each group after recruitment, the efficacy of each intervention, and the cost-effectiveness of telephone calls versus SMS reminders on re-recruitment.

#### Statistical analysis

The database and foundation for analyses were established by recoding data in an Excel software (2013, Microsoft Corporation, Redmond, WA, USA) file and importing into the Statistical Package for Social Sciences software (SPSS Statistics version 23 for Windows, SPSS Inc., Armonk, NY, USA) and The R Project for Statistical Computing (R version 3.6.1). For intention-to-treat (ITT) analysis, the re-donation rate was calculated by dividing the number of participants who donated again during the follow-up period by the corresponding number of initially randomised donors. Because of the large disparity in the intervention received rates between the two interventional groups, the ITT result might have masked a true effect on the re-donation rate among those who received reminders as intended. Therefore, estimation of the effects of the interventions on inactive blood donors while accounting for compliance with assigned intervention was also conducted. Previous studies defined four compliance types on the basis of individuals’ treatment assignment status and potential treatment receipt status [30–32]. In this study, strict adherence to the intervention assignment meant that those in the control group did not receive any telephone call or SMS message; meanwhile, participants in the telephone group did not receive an SMS message and vice versa. Thus, in this case, there were no directly observed always-takers (defined as those who will always implement the treatment, regardless of the group to which they are assigned), nor defiers (defined as those who will not implement if assigned to the treatment group but will implement if assigned to the control group). The participants did include compliers (defined as those who will implement the treatment when assigned to the treatment group but will not implement if assigned to the control group) and those could still be never-takers (defined as those who will never implement, regardless of the treatment assignment). In other words, this was a one-sided non-compliance situation, with only compliers (who received the telephone or SMS reminders successfully in the intervention groups, and who were in the control group) and never-takers (who failed to receive the telephone or SMS reminders in the intervention groups) [33]. Therefore, the average treatment effect on the treated (ATT) were also estimated [33].

Chi-square test was used to identify statistical differences of the re-donation rate among groups and conduct paired comparisons between contact methods, and Bonferroni correction was applied. R Package “ATE” was used to estimate the ATT among compliers under the intervention and control conditions (random assignment was used as an instrumental variable that telephone or SMS group coded as 1, control group coded as 0; complier in the telephone or SMS group was coded as 1, never-taker and those in the control group were coded as 0).

The Kruskal-Wallis test was used to determine whether the re-donation intervals within 30, 90, 180, 270 and 365 days were affected by different reminders, and Mann-Whitney U test was applied for comparisons of two groups. Binary logistic regression analyses were adopted to identify associations of donor characteristics with donor return behaviour to determine the best predictors of future donation; odds ratio (ORs) and 95% confidence intervals (CIs) were calculated. The incremental cost-effectiveness ratio (ICER) was applied to compare the cost-effectiveness of telephone calls and SMS reminders. All hypothesized differences were considered statistically significant if the *P*-values from two-tailed tests were < 0.05.

### Ethics considerations

All procedures were reviewed and approved by Institutional Review Board of the Guangzhou Blood Center. The registration ID for this study on ClinicalTrial.gov is: NCT03366441. This study is reported according to the CONSORT guidelines (Additional file 2).

## Results

### Study participants

Twenty-nine donors in the telephone group, 37 in the SMS group, and 26 in the control group found to have donated blood before the day of recruitment. They were all excluded and replaced by an equal number of matched participants in the same tier. In the telephone group, 40.0% of the participants were successfully interviewed, 20.8% could not be reached due to an incorrect number, 36.1% did not answer the phone and 3.1% refused to participate. In the SMS group, 79.3% participants received the message successfully, and 20.7% did not (successful intervention rates in telephone and SMS groups: 40.0% vs. 79.3%, *P* < 0.001).

### Interventions

#### Effects of interventions on donor return

For ITT analysis, the re-donation rates were 8.1% (*n* = 322) in the telephone group, 8.5% (*n* = 337) in the SMS group, and 7.4% (*n* = 291) in the control group. Chi-square test showed no significant difference in the re-donation rates among the three groups (*P* = 0.154). The re-donation rates within compliers in the telephone and SMS groups were 11.7% (185/1583) and 8.6% (270/3142), respectively. Table 1 shows the ATT estimation results that among those who received the intervention successfully, the telephone call was estimated to significantly increase re-donation by 2.3 percentage points compared to SMS reminder, and by 6.0 compared to no intervention.

**Table 1:** Average treatment effect on the treated estimations among three groups

	Point Estimate	Standard Error	95% CI	*Z*	*P*
Telephone vs. SMS	0.023	0.008	0.007, 0.038	2.885	0.004
Telephone vs. Control	0.060	0.009	0.041, 0.078	6.363	< 0.001
SMS vs. Control	0.004	0.011	−0.017, 0.025	0.372	0.710

#### Interaction between covariates and receipt of reminders on donor return

Table 2 summarizes the basic information of the reactivated donors. Logistic regression analysis showed that donors who were older, those with a larger donation frequency before recruitment, or those who accepted the interventions successfully were more likely to re-donate (Table 3). Table 4 indicates the associations of re-donation and donors who were successfully interviewed, received the message and were in the control group. Donor return behaviour was positively associated with receiving reminders, being male, being of older age, and having a previous donation history. Among those participants who were successfully contacted, older donors (1.02, CI: 1.00–1.04, *P* = 0.019) and those with a greater past donation frequency (1.08, CI: 1.03–1.13, *P* = 0.001) in the telephone group; as well as male donors (1.33, CI: 1.03–1.71, *P* = 0.028) and those with a greater past donation frequency (1.11, CI: 1.07–1.16, *P* < 0.001) in the SMS group were more likely to return.

**Table 2:** Summary of reactivated donors among all participants in the three groups

	Telephone group − 1	SMS group − 2	Control group −3	*P*		
				1 vs. 2	1 vs. 3	2 vs. 3
Gender						
Male	164 (51.1)	186 (55.2)	158 (54.3)	0.292	0.428	0.822
Female	157 (48.9)	151 (44.8)	133 (45.7)			
Age, years^a^	36.8 ± 8.0	37.0 ± 8.0	37.3 ± 7.5	0.873	0.203	0.150
Previous donations, n^a^	4.8 ± 6.5	4.5 ± 3.5	4.4 ± 3.1	0.250	0.167	0.597
Type of re-donation						
Whole blood	306 (95.3)	328 (97.3)	282 (96.9)	0.003	0.363	0.388
Apheresis platelet	15 (4.7)	9 (2.7)	9 (3.1)			
Additional donations, n						
1	291 (90.7)	305 (90.5)	269 (92.4)	0.213	0.318	0.752
≥ 2	30 (9.3)	32 (9.5)	22 (7.6)			

Data presented as no. (%), unless otherwise stated. ^a^Data presented as mean ± standard deviation

**Table 3:** Logistic regression analysis of associations of groups, donor characteristics, and intervention status with re-donation among all participants

	OR (95% CI) 1 vs. 2 vs. 3	OR (95% CI) 1 vs. 2	OR (95% CI) 1 vs 3	OR (95% CI) 2 vs. 3
Group				
Telephone call-1	0.92 (0.76, 1.11)	1.10 (0.93, 1.31)	0.80 (0.55, 1.00)	–
SMS-2	0.83 (0.66, 1.04)	reference	–	1.17 (0.89, 1.55)
Control-3	reference	–	reference	reference
Gender				
Male	1.14 (0.99, 1.31)	1.13 (0.96, 1.33)	1.10 (0.93, 1.30)	1.20 (1.02, 1.41)^*^
Female	reference	reference	reference	reference
Age	1.03 (1.02, 1.04)^**^	1.03 (1.02, 1.04)^**^	1.03 (1.02, 1.04)^**^	1.03 (1.02, 1.04)^**^
Donation history	1.08 (1.06, 1.10)^**^	1.11 (1.08, 1.13)^**^	1.07 (1.05, 1.09)^**^	1.06 (1.04, 1.08)^**^
Status of intervention^a^				
Successful	1.56 (1.30, 1.88)^**^	1.56 (1.29, 1.88)^**^	2.01 (1.60, 2.55)^**^	1.02 (0.77, 1.35)
Failed	reference	reference	reference	reference

Coding: Telephone call = 1, SMS = 2, Control = 3; Male = 1, Female = 2; Status of intervention (Successful) =1, (Failed/Control) = 2

^a^: Referred to those who accepted the interventions successfully; *: *P* < 0.05; **: *P* < 0.001

**Table 4:** Logistic regression analysis of associations of groups and donor characteristics with donor reactivation among those who were successfully contacted

	OR (95% CI) 1 vs. 2 vs. 3	OR (95% CI) 1 vs. 2	OR (95% CI) 1 vs 3	OR (95% CI) 2 vs. 3
Group				
Telephone call-1	1.63 (1.34, 1.98)^**^	1.33 (1.09, 1.63)^**^	1.62 (1.33, 1.97)^**^	–
SMS-2	1.20 (1.00, 1.43)^*^	reference	–	1.20 (1.01, 1.43)^*^
Control-3	reference	–	reference	reference
Gender				
Male	1.25 (1.07, 1.45)^*^	1.28 (1.05, 1.56)^*^	1.19 (0.99, 1.44)	1.24 (1.04, 1.48)^*^
Female	reference	Reference	reference	Reference
Age	1.02 (1.01, 1.03)^**^	1.01 (1.00, 1.03)^*^	1.03 (1.02, 1.04)^**^	1.02 (1.10, 1.03)^**^
Donation history	1.06 (1.04, 1.08)^**^	1.10 (1.06, 1.13)^**^	1.05 (1.03, 1.07)^**^	1.06 (1.04, 1.08)^**^

Coding: Telephone call = 1, SMS = 2, Control = 3; Male = 1, Female = 2

*: *P* < 0.05; **: *P* < 0.001

#### Impact of reminders on time to return

Table 5 shows the 1-year re-donation intervals for the three groups. The Kruskal-Wallis test revealed significant differences in the time to re-donation only within a 180-day interval among the three groups (*P* = 0.023), but not within the other intervals (data not shown). The Mann-Whitney U test indicated that participants returned to donate sooner in the SMS group (76.7 ± 50.9) than those in the control group (90.9 ± 51.2) within the 180-day interval (*Z* = 2.730, *P* = 0.006).

**Table 5:** Donors’ 1-year re-donation intervals (in days) among the three groups (mean ± SD)

	Intervals for reactivated donors who were enrolled	Intervals for reactivated donors who were successfully contacted
Telephone group	1 to 365 (157.4 ± 102.8)	1 to 365 (163.3 ± 95.8)
SMS group	1 to 365 (141.5 ± 102.7)	1 to 365 (155.5 ± 101.4)
Control group	1 to 365 (151.7 ± 99.2)	Not applicable

#### Cost-effectiveness of telephone and SMS reminders

The ICERs for the telephone and SMS reminders were evaluated from the bottom-up approach. In the telephone group, all interviews were completed in 7694 min, while recruiters waited on hold three times for a total of 7131 min. The hourly pay for one recruiter was RMB¥60 in the Guangzhou Blood Center, and therefore, the cost was RMB¥1 for a recruiter to work for 1 min. The telephone merchant charged RMB¥0.22 for the first 3 min and RMB¥0.11 for every 1 min thereafter for one call, whereas answering a call was free of charge. Thus, it cost RMB¥678.6 totally for 1583 calls. The average cost per participant in this group was:

$$ \frac{\left(7694+7131\right)\times 1+678.6}{3960}=\mathrm{RMB}\yen 3.9 $$

In the SMS group, it took 5 min for one recruiter to send all the messages. The system maintenance cost for the automatic message sending system is RMB¥2523 per year, and the SMS operator charges RMB¥0.05 for each message successfully sent. Thus, the total cost was RMB¥157.1 for sending 3142 messages successfully. The average cost per participant in this group was:

$$ \frac{5+2523+157.1}{3960}=\mathrm{RMB}\yen 0.7 $$

Table 6 presents the ICER estimation results, which indicated that the SMS reminder was more cost-effective than the telephone call.

**Table 6:** Incremental cost-effectiveness ratio estimations of telephone and SMS groups

	Cost per participant (C) (RMB¥)	Effectiveness (E) (%)	ΔC	ΔE	Ratio (ΔC/ΔE)
Control group	–	7.4	–	–	–
Telephone group	3.9	8.1	3.9	0.007	557.1
SMS group	0.7	8.4	0.7	0.010	70

### Self-reported reasons for blood donation deferral

**Fig. 2:**
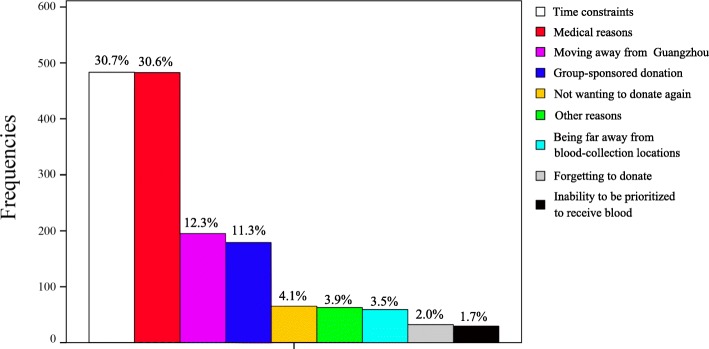
Distribution of self-reported reasons for deferral

The distribution of self-reported reasons for deferral is shown in Fig. 2. Those who reported time constraints were more likely to return after a phone call reminder than those who claimed other deterrents (14.2% vs. 10.6%, *P* = 0.037). Medical reasons included multiple different causes (Table 7). Donors who believed they had an inadequate health status were not able to reveal more specific details. Group-sponsored donation is a special form of donation in China, which is defined as blood donation organized by universities, companies, governmental agencies and any other groups. Seventy-seven (43.0%) former donors who had donated during a group-sponsored event had not donated again because they had missed the blood donation activity organized by their affiliation, 66 (36.9%) did not donate again because their affiliations stopped organizing the blood donation activity, and 36 (20.1%) reported that they did not re-donate because there is no “quota” for them to donate. Donors who simply did not want to donate again did not provide more information even when they were further asked about the reasons. Among the “other reasons”, “adverse reaction” was reported by the highest percentage of donors (23/60, 38.3%). Table 8 compares the willingness of former donors to re-donate and the actual re-donation rates according to the different self-reported deterrents to re-donation.

**Table 7:** Medical reasons for the lack of re-donation reported by donors who were successfully contacted by telephone (n, %)

Reasons	n, %
Self-perception of inadequate health status	197 (40.8)
Pregnancy/lactation	124 (25.7)
Confirmed diagnosis of severe disease	43 (8.8)
Self-perception as being too old to donate	42 (8.7)
Becoming unhealthy after blood donation	33 (6.8)
Confirmed diagnosis of anaemia	23 (4.8)
Other temporary reasons for deferral^a^	21 (4.3)

^a^Other temporary reasons for deferral included reasons such as ineligible weight, menstrual disorder etc

**Table 8:** Summary of willingness to re-donate and re-donation status of donors contacted by telephone according to the different reported reasons for deferral (*n*, %)

	Total	Willing to re-donate	Not willing to re-donate	Uncertain	Actually re-donated
Time constraints	485	444 (91.5)	5 (1.0)	36 (7.4)	69 (14.2)
Medical reasons	483	266 (55.1)	150 (61.0)	67 (13.9)	37 (7.7)
Self-perception of inadequate health status (unrelated to donation)	197	125 (63.5)	38 (19.3)	31(15.7)	20 (8.7)
Pregnancy/lactation	124	101(81.4)	7 (5.6)	16 (12.9)	5 (4.0)
Self-perception as being too old to donate	42	14 (33.3)	14 (33.3)	14 (33.3)	3 (7.1)
Becoming unhealthy after blood donation (related to donation)	33	7 (21.2)	22 (66.7)	4 (12.1)	3 (9.1)
Moving away from Guangzhou	195	55 (28.2)	136 (69.7)	4 (2.1)	18 (9.3)
Group-sponsored donation	179	160 (89.4)	7 (3.9)	12 (6.7)	26 (14.5)
Not wanting to donate again	65	25 (38.5)	21 (32.3)	19 (29.2)	6 (7.7)
Being far away from blood collection locations	56	53 (94.6)	1 (1.8)	2 (3.6)	12 (21.4)
Forgetting to donate	31	30 (96.8)	1 (3.2)	0 (0.0)	6 (19.4)
Inability to be prioritized to receive blood	27	8 (29.6)	15 (55.6)	4 (14.8)	4 (14.8)
Adverse reaction	23	3 (12.5)	2 (8.7)	18 (75.0)	1 (4.2)

## Discussion

Blood donor retention and re-enrolment are critical for the collection of a sufficient blood supply but are challenges in clinical practice. The use of a stratified, randomised trial design in the present study allowed us to test the efficacy and cost-effectiveness of telephone call and SMS message reminders for prompting inactive blood donors to donate blood again. Altruism, as a genuine part of human nature, has been found to be the most frequent motivator driving people to donate blood [23–25]. In the SMS group, inactive donors received a short text message on their cell phones that contained an altruistic appeal that emphasized how they could “save a life”, and in the telephone group, in addition to the altruistic appeal, inactive donors were also questioned about the reasons why they stopped donating and provided corresponding solutions according to their answers. ITT analysis showed that the differences in the re-donation rates among all participants in the three groups were not statistically significant. ATT estimations revealed that among those who received the interventions successfully, the telephone call was more effective than the SMS reminder or no intervention. Our results also showed that donor reactivation was positively associated with receiving reminders. Moreover, participants in the SMS group returned to donate sooner than control participants (*P* = 0.006) within 6 months and based on the calculated ICERs, SMS reminders were more cost-effective than telephone calls. In summary, interventions to promote inactive donors’ return to give blood are appropriate.

As mentioned above, an altruistic (‘save a life’) message was used in the SMS group in this study, because that help-seeking message can evoke empathy, create positive emotional feelings in the reader and connect them to the recipient of their help, which eventually increases helpful behaviour [34]. Nonetheless, it has been proven that blood donation is driven by multiple motives [22, 23, 35], and the results of the present study suggest that a message with an altruistic appeal might not be strong enough to prompt action among donors. Gemelli et al. found that sending a personalized post-donation message was effective for retaining donors [14]. Notably, the SMS messages sent via the Guangzhou Blood Center automatic message sending system are all personalized. After donation each donor receives a message including his/her name, blood type and blood test results confirming their eligibility to donate (donors who are ineligible are informed by phone call). Therefore, the message sent for the purpose of recruiting inactive blood donors becomes personalized if sent via the automatic message sending system, which might increase the re-donation rate. Moreover, male donors or those with a greater past donation frequency were more likely to return after they received a message with an altruistic appeal, and thus, SMS reminders can be used to target these donors. The SMS reminder is overall an effective and convenient strategy for reactivating inactive donors, with the additional advantage of being cost-effective. After the study period, SMS messages were also sent to those who could not be reached in the telephone group and those in the control group.

Although the successful contact rate for the telephone group was much lower than that for the SMS group, the donors in the telephone group were more likely to return once they received the call successfully. Moreover, the effect was greater on those with a higher past donation frequency and older donors. Previous studies proved that the number of previous returns of a donor is positively associated with future return [6]. As more donations are made, the perception of oneself as a donor becomes internalized and serves as a motivating force for repeat donation [36]. Blood donors who gave more than 4 donations a year considered blood donation as an act of altruism and promised to continue donating blood in the absence of benefits and rewards [37]. Therefore, altruistic appeal, by either telephone or SMS reminder, was effective for these donors.

In this study, we not only used an altruistic appeal but also communicated with the donors to better understand their self-reported reasons for blood donation deferral. The altruistic appeal via the telephone call had a significantly greater positive effect on those who reported time constraints than on those who claimed other deterrents kept them from donating again, and in multiple studies, time constraints were the most frequently stated factor preventing donors from continuing to donate blood [21, 38, 39]. One study found that people believed that spending time on blood donation had no more or less value than any other moment in their day [17]. They might have a positive attitude towards a request for blood donation, but did not take corresponding action due to a lack of urgency or motivation [40]. When we mentioned the idea of “saving a life” to emphasize the urgency of the need as well as revisit their original motivation for donating, they returned to donate. Therefore, blood donation agencies should make efforts to minimize the time required for donation, to implement more extensive and flexible opening hours, and also to convince donors of the importance of donation.

An altruistic appeal could not effectively reactivate those who reported medical reasons for their donation deferral. Self-perceived inadequate health status, adverse reaction to blood donation, and becoming unhealthy after blood donation represented particular barriers to blood donation and seem to have similarities in China and other countries. In the Chinese traditional culture, people believe that blood is vital to human life (the Mother of Qi) and loss of blood equates to ruining one’s constitution [41]. Once donors experience adverse events or even they simply feel tired after donation, they likely deem that they experienced substantial detrimental effects or long-term consequences from blood donation. In addition, some donors may have mentioned medical barriers as a “false” reason that is more socially acceptable than stating that they do not have time [38]. To develop a strategy to recruit these donors, more specific psychological research should be carried out.

Blood donors in China typically fit into one of two types: those who spontaneously donate at blood collection sites, and those who donate through a group donation. State-owned and state-run enterprises and governmental agencies will compensate workers with either subsidies (small amounts of money for nutritional supplementation) or a few days off (with or without paid vacation), and hence, some groups will limit the number of donors. In light of these findings, the objectives of group donors for blood donation might include a combination of motives, such as modestly self-serving incentives, instead of pure altruism. In addition, with the convenience of the donation process occurring at ones’ place of work, donors do not perceive blood donation as time-consuming or something that must be done too far away. According to these factors, individuals rarely donate again at blood collection sites, if their group stops organizing a blood donation activity or has no quota. In the present study, most group donors refused to donate at blood collection sites because they thought it too inconvenient, but they would donate again at their group.

Our study has some limitations. First, we only used an altruistic appeal in the SMS group, and altruism might not be the main driver for blood donation. Further research using different messages to re-recruit donors is needed to determine which type of message is the optimal intervention. Secondly, telephone and SMS reminders are different interventions, and thus, we cannot determine precisely which factor prompted the donors to return. Future research that explores how inactive donors interact with the interventions is needed to determine which factor is more effective. Moreover, even with delivery confirmation receipts, as available in newer smartphones, one cannot know with certainty that messages were read and understood. In addition, just as we mentioned above, donation records from outside of Guangzhou could not be confirmed. Therefore, the donation frequency history for inactive donors might not be correct, and the re-donation rate, especially for those who cited moving out of Guangzhou as a deterrent, might be underestimated. Those who donated blood outside Guangzhou were cases lost to attrition, but in this randomised controlled trial, the attrition rates among the three groups were believed to be nearly equal. The response rate of telephone group was low (40%), but since the rate at which lapsed blood donors answered telephone interviews from Guangzhou Blood Center was 35–45% [27], the response rate in the present study was close to the real percentage.

## Conclusions

In conclusion, inactive blood donors may be encouraged to re-donate after receiving telephone calls or SMS reminders. Telephone calls may be more effective than SMS messages for reactivating inactive donors, but the effectiveness of each method must be weighed with the corresponding costs. More detailed studies are needed to evaluate the effects of the contents of the SMS message for reactivating inactive donors. Moreover, future studies should also focus on strategies for re-recruiting those who stopped donating because of a self-perception of inadequate health status.

## Supplementary information

**Additional file 1.** Interview script and examples of responses. Contents are the recruitment message scripts of the telephone groups.

**Additional file 2.** CONSORT 2010 checklist of information to include when reporting a randomised trial. Contents are the CONSORT 2010 checklists of this study.

## Data Availability

The datasets used and analysed during the current study are available from the corresponding author on reasonable request.

## Abbreviations

ITT: Intention-to-treat
ATT: Average treatment effect on the treated
ICER: Incremental cost-effectiveness ratio
SMS: Short message service
