# Supplementary material for: Effective methods for reactivating inactive blood donors: a stratified randomised controlled study
Source: BMC Public Health. 2020 Apr 10;20:475. doi: 10.1186/s12889-020-08594-9 (PMC7147048; doi:10.1186/s12889-020-08594-9)
Supplement: Supplementary file 1 — Additional file 1. Interview script and examples of responses. Contents are the recruitment message scripts of the telephone groups. [file 12889_2020_8594_MOESM1_ESM.docx]

**Interview script and examples of responses**

Contents below were all translated from Chinese

**Interview script**

- Good morning/afternoon! May I speak to (donor’s name)?

- Hello Mr./Ms. (donor’s name), this is (interviewer’s name) from Guangzhou Blood Center. I’m calling to thank you for your blood donation, which has saved someone’s life. We noticed that you have stopped donating since (date) though; could you tell me the main reason why you stopped?

*If the donor reports permanent deterrent:*

- Thank you again for your donation. I hope you have a good day, goodbye.

*If the donor reports a temporary deterrent:*

- (According to the answers of the donor, interviewers would provide the corresponding explanations/supports/information). There are always patients waiting for blood, and every donation offers them hope. Are you willing to donate again when you are available?

*If yes:*

- Thank you for your participation and support, I hope you have a good day, goodbye.

*If no or uncertain:*

- Did you know that one donation would save three lives? Therefore, I hope you will reconsider it soon. Thank you for your participation. I hope you have a good day, goodbye.

**Examples of responses.**

Time constraints

- We can find the nearest blood collection sites and share their location with to save your time. There are always patients waiting for blood, and every donation offers them hope. Therefore, are you willing to donate again when you are available?

Moving away from Guangzhou

- There are always patients waiting for blood, in Guangzhou and elsewhere, and every donation offers them hope. Therefore, are you willing to donate again in your city of residence or any time that you return to Guangzhou?

Self-perception of inadequate health status

- We are sorry to hear that, and we hope you can feel better soon. There are always patients waiting for blood, and every donation offers them hope. Therefore, are you willing to donate again when you are available?

Pregnancy/lactation

- Congratulations. You can donate again once you have completed lactation. There are always patients waiting for blood, and every donation offers them hope. Therefore, are you willing to donate again when you are available?

Self-perception as being too old to donate

- The age limitation for blood donation is 55 years, and those who have donated at least 3 times before age 55 years can extend their donations until age 60 years in China. In some countries, there is no restrictions on donor age. In addition, blood donation could help boost metabolism^1^, reduce the risk of cancer by iron reduction^1^, and reduce the risk of heart disease^2^. Moreover, there are always patients waiting for blood, and every donation offers them hope. Therefore, are you willing to donate again when you are available?

Group-sponsored donation

- You can donate at your nearest blood collection site, because there are always patients waiting for blood, and every donation offers them hope. Therefore, are you willing to donate again when you are available?

Inability to be prioritized to receive blood

- We are sorry for what happened to you. Before 2014, in Guangzhou, when a donor or donor’s family members needed an elective surgery, they had to either wait on a waiting list until blood products were ready or had to receive family/replacement donation. We know that this issue bothers many donors. Therefore, since 2014, the government of Guangzhou has stipulated that a donor and the donor’s family members have priority for receiving blood when needed, even during an elective surgery. We are sorry again. There are always patients waiting for blood though, and every donation offers them hope. Therefore, are you willing to donate again when you are available?

Becoming unhealthy after blood donation/adverse reaction

- We are sorry that you did not feel well after donating blood. One possible reason is that you did not sleep well the night before donating^3^. Please consider trying donation again, because there are always patients waiting for blood, and every donation offers them hope. Therefore, are you willing to donate again when you are available?

1. Edgren G, Reilly M, Hjalgrim H, Tran TN, Rostgaard K, Adami J, et al. Donation frequency, iron loss, and risk of cancer among blood donors. Journal of the National Cancer Institute. 2008;100(8):572-9.
2. Salonen JT, Tuomainen TP, Salonen R, Lakka TA, Nyyssonen K. Donation of blood is associated with reduced risk of myocardial infarction. The Kuopio Ischaemic Heart Disease Risk Factor Study. American journal of epidemiology. 1998;148(5):445-51.
3. Masser BM, White KM, Terry DJ. Beliefs underlying the intention to donate again among first-time blood donors who experience a mild adverse event. Transfusion and apheresis science : official journal of the World Apheresis Association : official journal of the European Society for Haemapheresis. 2013;49(2):278-84.
